# Case Report: Diagnostic and therapeutic reflections on one case of greater omental adhesion misdiagnosed as peritoneal metastasis following rectal cancer surgery

**DOI:** 10.3389/fonc.2026.1738387

**Published:** 2026-05-04

**Authors:** Chaoming Li, Taojun Liang, Chenghuan Yang, Xihui Chong, Binbin Du, ZhengPeng Qian, Haixia Li

**Affiliations:** 1 Longnan First People’s Hospital, Longnan, China; 2 Gansu Provincial Hospital, Lanzhou, China

**Keywords:** intra-abdominal adhesion, misdiagnosis, multidisciplinary team (MDT), peritoneal metastasis, rectal cancer

## Abstract

**Objective:**

To investigate the differential diagnostic criteria and decision-making logic for distinguishing benign intra-abdominal adhesions from peritoneal metastasis following preoperative radiotherapy and surgical resection for rectal cancer, thereby providing clinical reference for managing similar atypical peritoneal lesions.

**Methods:**

We conducted a retrospective analysis of clinical data from a 57-year-old patient with stage pT4bN1cM0 rectal cancer who received postoperative radiotherapy at our institution. The patient was initially suspected of having peritoneal metastasis based on an abnormally enhanced nodular lesion in the right lower anterior abdominal wall. We systematically reviewed the diagnostic and therapeutic process, multidisciplinary team (MDT) decision-making, and pathological diagnosis, and analyzed the causes of misdiagnosis, differential diagnostic key points, and management strategies in conjunction with relevant literature.

**Results:**

The patient underwent laparoscopic exploration combined with abdominal wall lesion resection. Postoperative pathology confirmed greater omental adhesion with localized inflammatory reaction, effectively excluding malignant metastasis.

**Conclusions:**

Cross-sectional imaging has inherent limitations in the diagnosis of peritoneal lesions. For patients with suspected peritoneal lesions following rectal cancer surgery, comprehensive evaluation should integrate trauma history, tumor markers, clinical symptoms, and imaging characteristics. The patient had no evidence of extraperitoneal metastasis throughout the diagnosis and treatment, and the interval between the initial radical rectal cancer surgery and the detection of suspected peritoneal metastatic lesions was 2 years. Individualized diagnostic and therapeutic decisions guided by MDT, with appropriate application of laparoscopic minimally invasive exploration, can effectively improve diagnostic accuracy and avoid overtreatment or diagnostic delays.

## 1 Introduction

Colorectal cancer (CRC) remains one of the most prevalent and lethal malignancies of the digestive system, imposing a continuous burden on public health systems worldwide (1). Peritoneal metastasis (PM) represents a critical route of disease dissemination and substantially compromises patient prognosis: approximately 5%-15% of patients develop synchronous peritoneal metastasis, while 20% experience metachronous metastasis, with a median survival time of merely 6–7 months (2). Early and accurate differentiation between benign and malignant peritoneal lesions is therefore paramount for improving clinical outcomes. Since Professor Stephen Paget proposed the “seed and soil hypothesis” in 1889, peritoneal metastasis has garnered considerable scholarly attention (3).

Surgical resection occupies a pivotal position in the comprehensive management of CRC. However, this is accompanied by a rising incidence of postoperative intra-abdominal adhesions following rectal cancer surgery, which are closely associated with surgical trauma, ischemia-reperfusion injury, and infection, with clinical manifestations predominantly including abdominal pain and intestinal obstruction (4, 5).

Currently, cross-sectional imaging (contrast-enhanced CT/MRI) is recognized as the initial screening modality for peritoneal metastasis. Nevertheless, this approach exhibits substantial limitations, with both false-positive findings (fibrosis, inflammatory adhesions, postoperative changes, etc.) and false-negative results constituting well-documented clinical challenges (5, 6). Postoperative intra-abdominal adhesion following rectal cancer resection is a common complication of surgical trauma. The dual trauma from preoperative radiotherapy combined with surgery can lead to superimposed local inflammatory responses and fibrotic proliferation, whose imaging manifestations may be readily confused with the nodular enhancing lesions characteristic of peritoneal carcinomatosis (7, 8).

Although the limitations of imaging modalities have been extensively validated, there remains a paucity of concrete clinical case references regarding the differential diagnosis and therapeutic decision-making for atypical peritoneal nodules in high-risk stage rectal cancer patients with a history of combined radiotherapy and surgical trauma. This paper presents a retrospective analysis of one case in which a post-rectal cancer surgery patient receiving radiotherapy was initially suspected of having peritoneal metastasis but was subsequently confirmed to have greater omental adhesion. In conjunction with existing consensus guidelines, we analyze the causes of misdiagnosis, elucidate key points for benign-malignant differentiation, and clarify the logic underlying individualized diagnostic and therapeutic decision-making, with the aim of providing practical clinical reference and reducing the occurrence of similar diagnostic errors.

## 2 Case report

### 2.1 Patient information and initial presentation

A 57-year-old female was admitted to our institution on January 15, 2022, due to rectal cancer. Pre-admission contrast-enhanced abdominal CT indicated rectal malignancy located in the lower rectum (extraperitoneal rectum, Rb) with clinical staging of T4aN1M0. No evidence of extraperitoneal metastasis was found in the patient during the initial preoperative staging evaluation.

### 2.2 Treatment course

From January 22 to March 4, 2022, the patient received pelvic external beam radiotherapy (IGRT 6MV-X-ray, PGTV-DT 59.92 Gy/28 fractions, PTV-DT 50.4 Gy/28 fractions) with concurrent capecitabine chemotherapy. On April 2, 2022, neoadjuvant chemotherapy with the CAPEOX regimen was administered, followed by laparoscopic abdominoperineal resection for rectal cancer on April 29, 2022. The main operating port of this laparoscopic surgery was located at about 2 transverse fingers medial to the anterior superior iliac spine in the right lower abdomen. Postoperative pathology revealed moderately differentiated ulcerative adenocarcinoma of the rectum, staged as pT4bN1cM0 (AJCC staging). From June to August 2022, the patient completed 5 cycles of CAPEOX adjuvant chemotherapy, with regular follow-up showing no abnormalities thereafter.

### 2.3 Discovery of suspected lesion

On May 24, 2024,2 years after the initial radical surgery, follow-up contrast-enhanced abdominal CT revealed an abnormally enhanced nodular lesion in the right lower anterior abdominal wall, which was consistent with the location of the main operating port of the initial laparoscopic surgery, prompting readmission for further evaluation. The patient presented with good general condition, without symptoms of abdominal pain, distension, or intestinal obstruction, and maintained stable body weight.

### 2.4 Physical examination

Vital signs were stable. No superficial lymphadenopathy was palpable. The abdomen was flat and soft, without tenderness or rebound tenderness. Hepatosplenomegaly was absent. Shifting dullness was negative. Bowel sounds were normal.

### 2.5 Laboratory findings

CEA was 5.25 ng/ml and CA199 was 8.42 U/ml, both within normal range. Albumin was 33.51 g/L, indicating mild hypoalbuminemia. Complete blood count and hepatic/renal function tests showed no significant abnormalities.

### 2.6 Imaging studies

**Figure 1:**
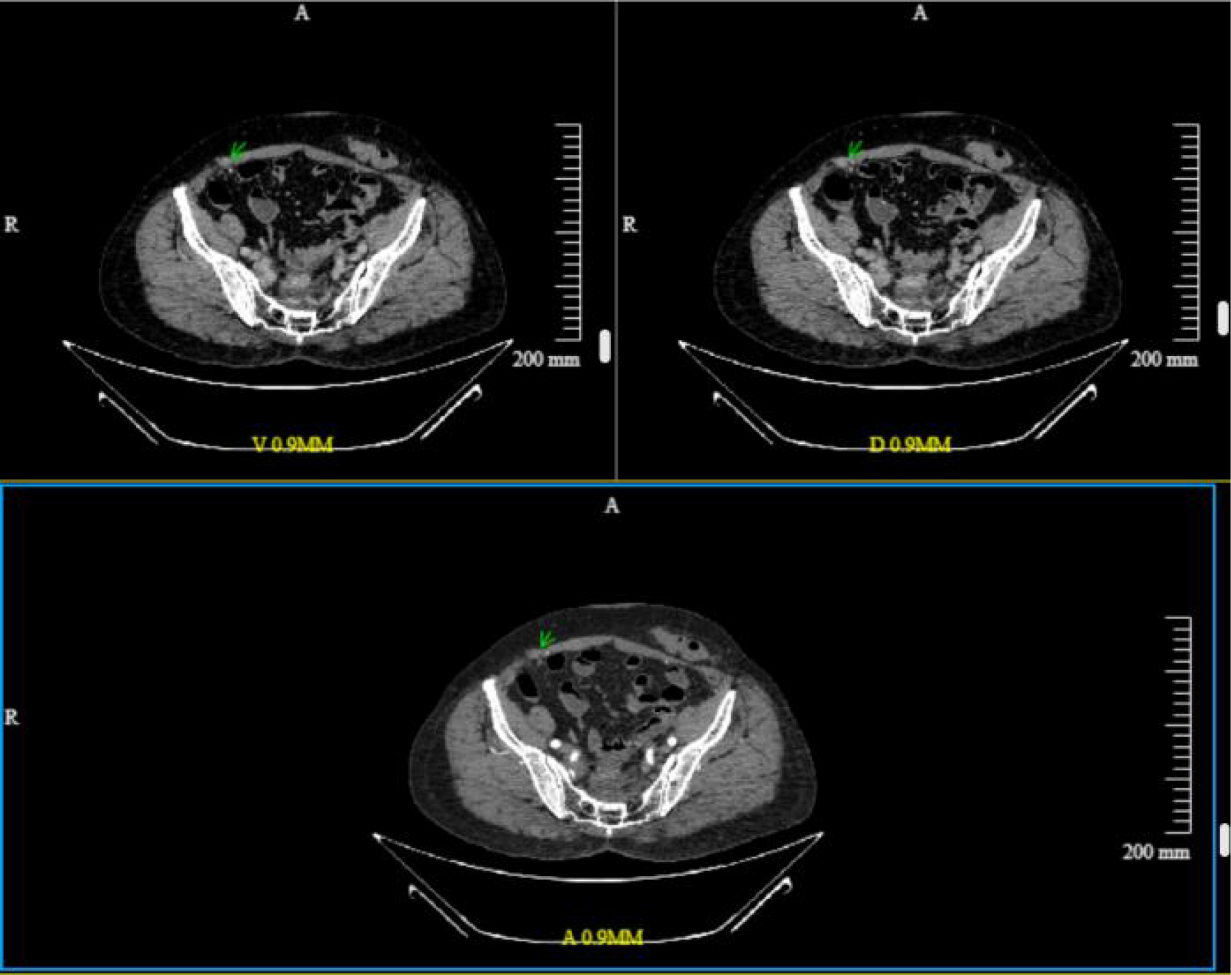
Contrast-enhanced CT scan in May 2024. The peritoneal nodule in the anterior abdominal wall (arrow) shows early enhancement in the arterial phase (lower middle) with visible intralesional vascularity, and persistent enhancement in the venous phase (upper left) and delayed phase (upper right), consistent with peritoneal metastasis.

**Figure 2:**
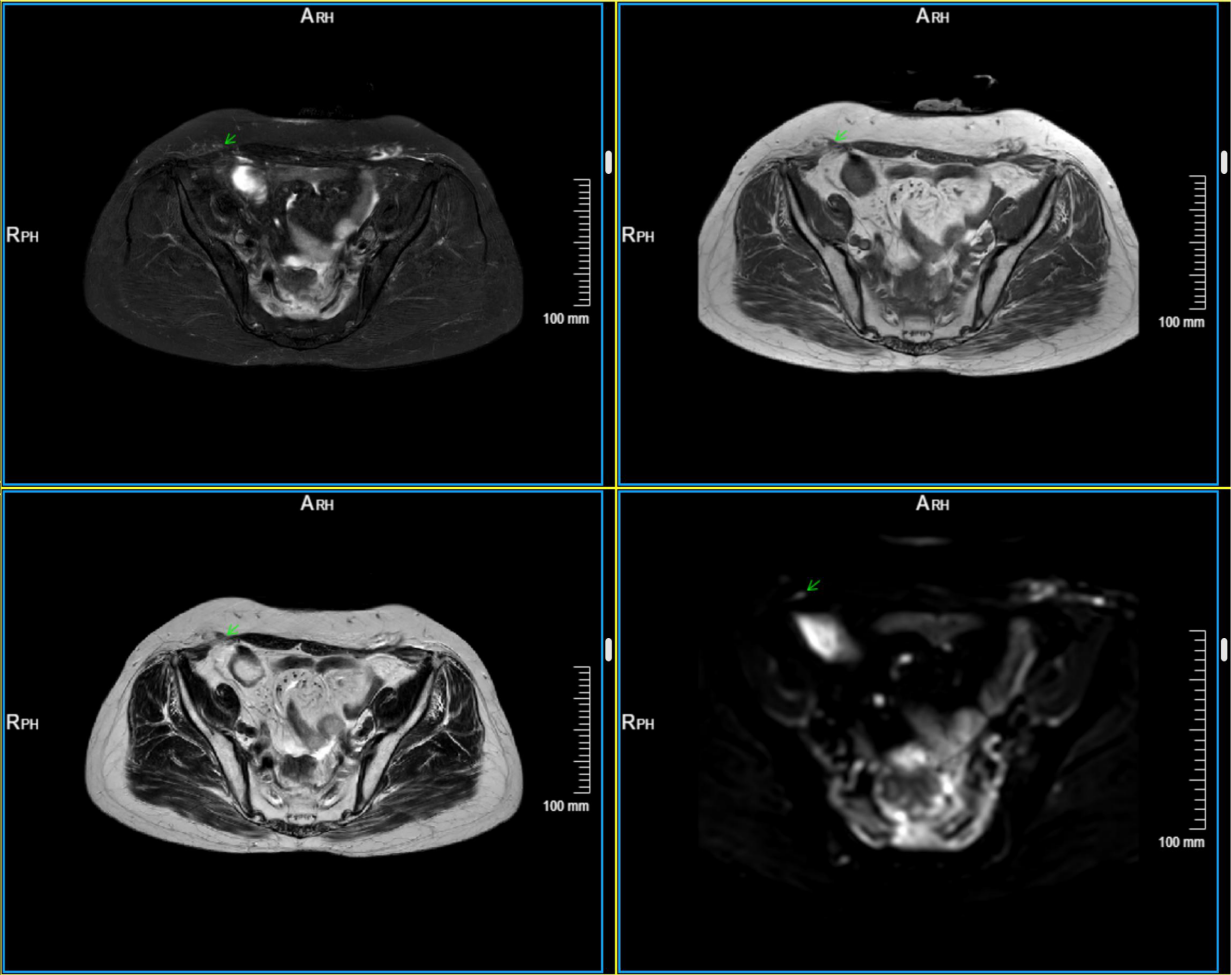
Contrast-enhanced MRI scan in May 2024. T2-weighted fat-saturated image (left upper) shows a hyperintense peritoneal nodule in the anterior abdominal wall (arrow). The lesion appears isointense to hypointense on axial T1-weighted image (right upper) with definite localization. Conventional T2-weighted image (left lower) is applied for comparison to exclude artifacts. Marked diffusion restriction is observed on diffusion-weighted imaging (DWI) (right lower).

Contrast-enhanced abdominal CT demonstrated an abnormally enhanced nodule (2.3 cm × 1.8 cm) in the right lower anterior abdominal wall, without ascites, bowel infiltration, or omental cake sign (**Figure 1**). Abdominal MRI (plain scan + contrast enhancement) revealed abnormal nodular enhancement, suggestive of possible metastasis (**Figure 2**). Electronic colonoscopy showed multiple colonic polyps and internal hemorrhoids, with no evidence of rectal tumor recurrence. Echocardiography and thoracic imaging were unremarkable. PET-CT was recommended as a supplementary examination for further differential diagnosis of the nodule due to its high diagnostic value for malignant lesions with glucose metabolism activity, but the patient refused to undergo the examination because of the high cost.

### 2.7 MDT discussion and decision-making

Following admission, a multidisciplinary team (MDT) meeting was convened, involving departments of medical oncology, gastrointestinal surgery, radiology, and pathology. The central controversy centered on surveillance observation versus laparoscopic exploration. Option one involved contrast-enhanced CT/MRI every 3 months, which avoids invasive procedures but may increase psychological burden for this high-risk patient (pT4bN1cM0) and carries potential risk of delayed intervention. Option two involved laparoscopic exploration with lesion resection, offering direct visualization of intra-abdominal conditions and pathological gold standard acquisition, but with minimally invasive surgery-related risks. Considering the patient and family’s explicit refusal of surveillance and their desire for prompt diagnostic clarification, the MDT ultimately selected laparoscopic exploration.

### 2.8 Surgical procedure and pathological findings

On June 4, 2024, the patient underwent laparoscopic exploration combined with abdominal wall lesion resection. Intraoperative exploration revealed local nodular formation due to adhesion between the right lower anterior abdominal wall and the greater omentum, without extensive peritoneal thickening, ascites, or bowel infiltration. The adhesive nodule and surrounding fibrofatty tissue were completely resected. Postoperative pathological examination (HE staining, 10×20 magnification) showed fibrofatty tissue with chronic inflammatory cell infiltration; no atypical cells or malignant tumor cells were observed. The final diagnosis was greater omental adhesion with localized inflammatory reaction.

### 2.9 Postoperative course

**Figure 3:**
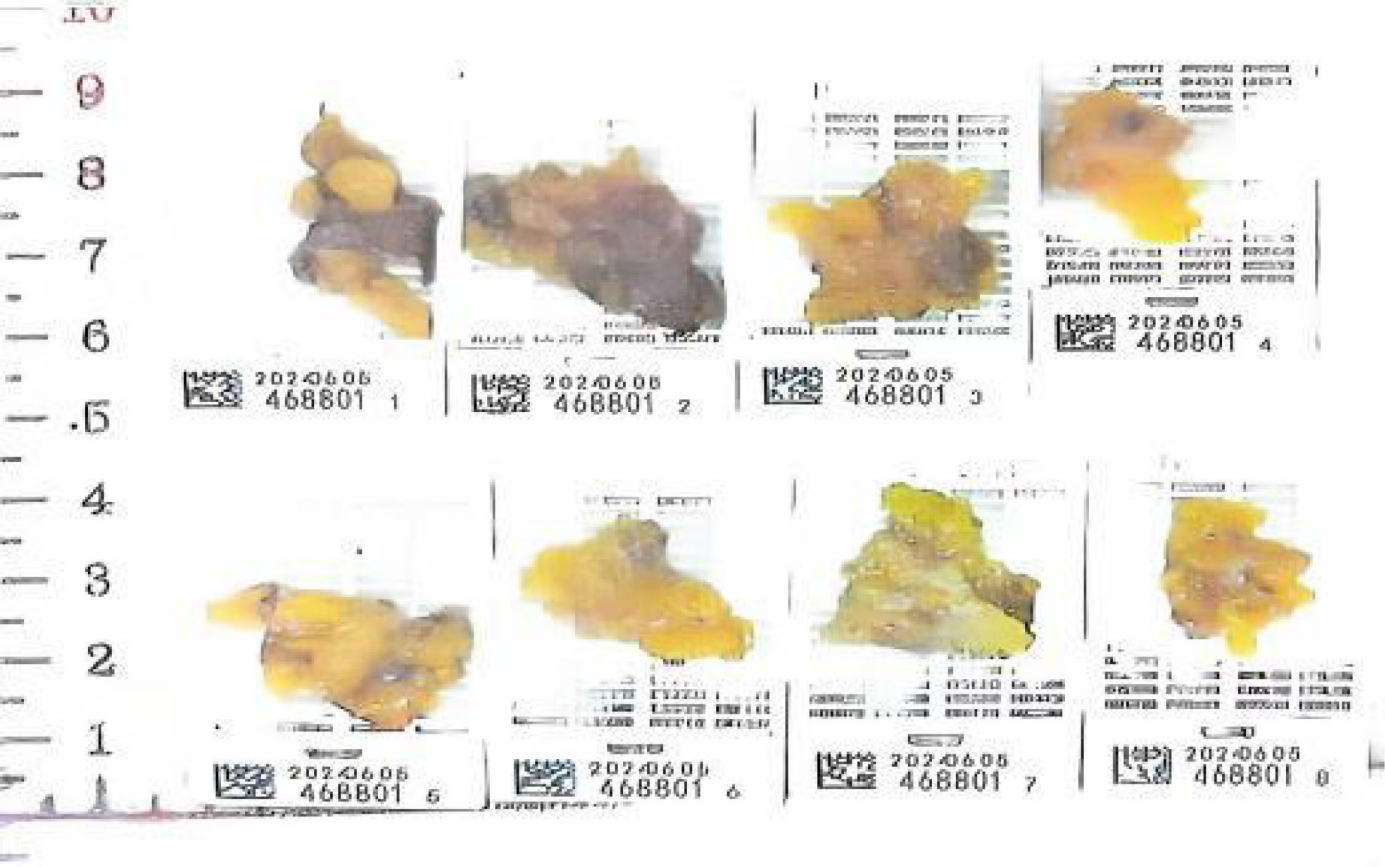
Surgical specimen obtained on June 4, 2024.

**Figure 4:**
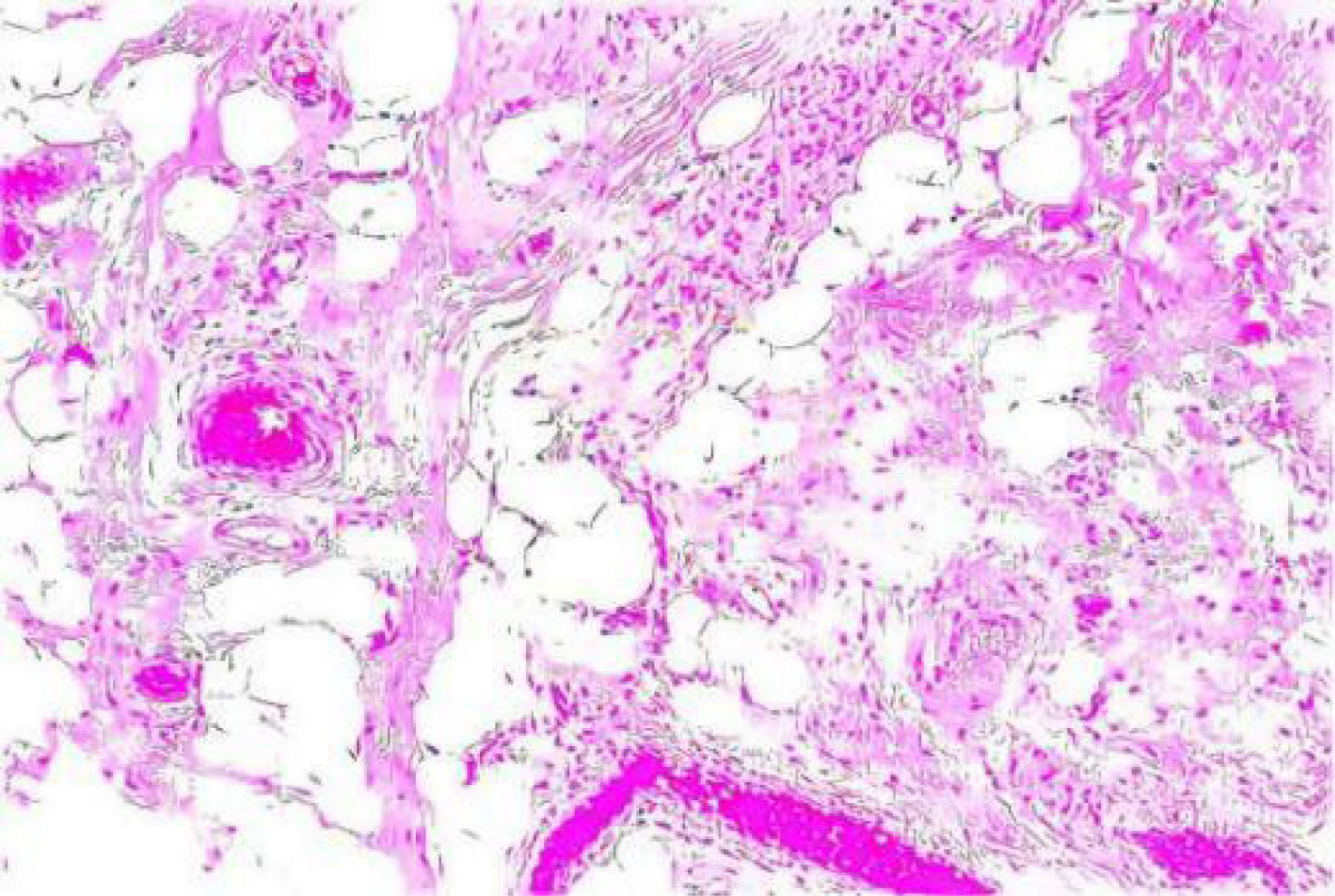
Postoperative pathological examination (10×20, hematoxylin-eosin staining).

The patient recovered smoothly without complications such as wound infection or intestinal obstruction. At one-year postoperative follow-up, no evidence of tumor recurrence or metastasis was observed. After multidisciplinary treatment (MDT) consultation, the possibility of secondary peritoneal malignancy was highly suspected, and diagnostic surgical intervention was recommended for pathological confirmation. Following exclusion of contraindications, laparoscopic exploration with abdominal wall lesion resection was performed on June 4, 2024. Postoperative pathology reported (**Figure 3**, **4**) resected tissue was fibrofatty tissue with localized inflammatory reaction, without evidence of atypical cells or malignant components.

## 3 Discussion

This case represents an atypical enhanced nodule formed by greater omental adhesion following dual trauma from preoperative radiotherapy combined with laparoscopic radical resection for rectal cancer, which was misdiagnosed due to imaging features closely mimicking peritoneal metastasis. This case highlights the inherent limitations of cross-sectional imaging in evaluating peritoneal lesions. Based on existing clinical consensus and the characteristics of this case, this article discusses four dimensions: imaging limitations, causes of misdiagnosis, key points for benign-malignant differentiation, and diagnostic-therapeutic decision-making logic, aiming to provide reference for clinical management of similar cases.

### 3.1 Inherent limitations of imaging in diagnosing peritoneal metastasis

Contrast-enhanced CT and MRI are first-line screening tools for peritoneal metastasis, with diagnostic criteria primarily including peritoneal thickening, nodular enhancement, ascites, and omental cake sign (6). However, multiple studies have confirmed that these examinations have inherent limitations of limited tissue resolution and insufficient specificity (2, 9).

Regarding false negatives, small metastatic lesions (<5 mm) manifest only as mild peritoneal enhancement and are easily missed. Regarding false positives, benign conditions such as postoperative adhesions, inflammatory fibrosis, post-radiotherapy tissue reactions, and tuberculous peritonitis can all form nodular or patchy enhanced lesions due to local fibrous and vascular proliferation, overlapping with the presentation of peritoneal metastasis (10). PET-CT has unique advantages in the differential diagnosis of benign and malignant peritoneal lesions: colorectal cancer generally exhibits overexpression of Hif1-α, which leads to active glucose uptake and metabolism of tumor cells, and for peritoneal nodules with a diameter of about 2 cm, PET-CT can effectively distinguish benign and malignant lesions by detecting the metabolic activity of the lesions with relatively high diagnostic accuracy. Although PET-CT can assist in differentiation through metabolic activity, its sensitivity for small, low-metabolic peritoneal lesions is only approximately 50% (11, 12). Diffusion-weighted MRI (DWI) can improve detection rates of small lesions through cell density differences, but has not yet been widely available at primary care institutions (13).

Given the above limitations, a multimodal combined diagnostic strategy has been established clinically: integrating MDT opinions, combining imaging, tumor markers, clinical symptoms, and medical history, with laparoscopic exploration for pathology when necessary. The diagnostic and therapeutic process of this case was conducted within this framework, and its value lies in providing insights for clinical implementation of existing strategies. In this case, the 2.3 cm × 1.8 cm nodule was the optimal indication for PET-CT, but the examination could not be completed due to the patient’s refusal for economic reasons, which also reflects the practical clinical dilemma in the application of high-value diagnostic techniques.

### 3.2 Imaging-based analysis of misdiagnosis causes in this case

The core reason for the suspected diagnosis of peritoneal metastatic carcinoma in this case was that benign adhesions induced by dual trauma from radiotherapy combined with surgery formed imaging features highly similar to metastatic lesions, with specific mechanisms as follows:

1. Preoperative pelvic external beam radiotherapy caused radiation-induced inflammation in local peritoneal and adipose tissues, with increased vascular permeability and neovascularization (14); postoperative laparoscopic procedures further induced greater omental adhesion to the abdominal wall (15). The superposition of these two traumatic effects promoted localized proliferation of fibrofatty tissue, forming nodular protrusions.
2. The “abnormal enhancement” on contrast-enhanced CT/MRI essentially reflects differences in tissue blood perfusion. The adhesive nodule in this case, due to inflammatory reaction accompanied by abundant neovascularization, showed increased blood perfusion, presenting as nodular enhancement on contrast-enhanced scanning, which was difficult to distinguish from tumor angiogenesis in peritoneal metastatic carcinoma, becoming the primary basis for imaging-based suspected diagnosis.
3. The adhesive nodule showed isolated and localized distribution, without presenting characteristic features commonly seen in intra-abdominal adhesions such as “bowel traction and angulation,” and imaging displayed only an isolated enhanced nodule, increasing the difficulty of differentiation (16, 17).
4. The nodule was located at the main operating port of the initial laparoscopic surgery, and port-site implantation is a common type of peritoneal metastasis after laparoscopic cancer surgery, which further increased the clinical suspicion of malignant metastasis and became an important auxiliary factor for the initial misdiagnosis.
5. Imaging diagnosis did not fully combine the patient’s dual trauma history of chemoradiotherapy and surgery at the initial stage, and interpreted imaging signs in isolation, which was a secondary factor leading to misdiagnosis.

### 3.3 Key points for benign-malignant differentiation of peritoneal lesions in rectal cancer patients with dual trauma history of radiotherapy plus surgery

Combining the characteristics of this case and existing literature (5–7, 10, 15, 18), we have organized the key points for benign-malignant differentiation of peritoneal nodular lesions in high-risk rectal cancer patients with dual trauma history of radiotherapy plus surgery. A practical differentiation standard is established from four dimensions: tumor markers, imaging features, clinical symptoms, and history characteristics, providing reference for clinical preliminary screening (**Table 1**).

**Table 1:** Key points for benign-malignant differentiation of peritoneal nodular lesions in rectal cancer patients with dual trauma history of radiotherapy plus surgery.

Differentiation dimension	Benign adhesion (Present Case)	Peritoneal metastatic carcinoma
Tumor Markers	CEA, CA199, etc. show no significant elevation	Often significantly elevated with progressive upward trend
Core Imaging Features	Isolated nodular lesion without ascites, omental cake sign, bowel infiltration/fixation, or extensive peritoneal thickening	May present as single or multiple nodules, often accompanied by ascites, omental cake sign, bowel infiltration/fixation; some cases show extensive peritoneal thickening
Imaging Enhancement Characteristics	Nodule enhancement is relatively homogeneous without low-density areas of tumor necrosis	Nodule enhancement is often heterogeneous, frequently with central low-density necrotic areas
Clinical Symptoms	No specific symptoms; no significant weight loss; no abdominal pain, distension, or intestinal obstruction	May present with progressive weight loss, abdominal pain, distension; intestinal obstruction and refractory ascites in advanced stages
Medical History and Disease Course	Clear history of dual trauma (radiotherapy + surgery); nodule size shows no significant change in short term; the nodule may be located at the surgical operating port with an interval of ≥2 years from the surgery	No specific inflammatory or traumatic history; nodules often show progressive enlargement with rapid disease progression; port-site lesions have a short interval with surgery and progressive enlargement

### 3.4 Diagnostic-therapeutic decision-making logic for atypical peritoneal lesions in high-risk rectal cancer patients after surgery

The management of suspected peritoneal lesions after rectal cancer surgery, particularly in high-risk staged patients such as pT4/N2, presents a core challenge in balancing “avoiding overtreatment” with “preventing diagnostic delay.” Based on the diagnostic and therapeutic experience of this case and existing literature, such decision-making can reference the following stratified approach:

Initial Screening Stratification: Risk stratification is performed based on tumor markers, imaging features, and clinical symptoms. Patients with significantly elevated tumor markers (19, 20), typical imaging presentation (21, 22), or accompanied by ascites or intestinal obstruction (23) are highly suspected of peritoneal metastasis and should directly undergo evaluation for anti-tumor treatment indications. Patients with normal tumor markers, isolated atypical nodules, and no specific symptoms have a higher probability of benign lesions and require further decision-making. For such patients, PET-CT should be prioritized as a supplementary examination if the patient’s economic condition permits, to improve the preoperative differential diagnosis accuracy. If peritoneal metastasis is confirmed, cytoreductive surgery combined with hyperthermic intraperitoneal chemotherapy (CRS+HIPEC) or systemic chemotherapy combined with targeted therapy should be selected based on the Peritoneal Carcinomatosis Index (PCI) and performance status. If benign lesions are confirmed, no anti-tumor treatment is required; routine follow-up is sufficient, with patients informed of adhesion-related risks (such as intestinal obstruction and chronic abdominal pain).For high-risk patients who refuse PET-CT or close follow-up, laparoscopic minimally invasive exploration is the first-choice diagnostic method, which can obtain the pathological gold standard while minimizing surgical trauma and is the most effective way to resolve the dilemma of imaging differential diagnosis.

## 4 Conclusion

This case once again confirms the inherent limitations of cross-sectional imaging in diagnosing peritoneal lesions, Notably, this case is characterized by no extraperitoneal metastasis, a 2-year interval between the initial surgery and nodule detection, and the nodule located at the main operating port of laparoscopy, which is a typical clinical scenario that is easily misdiagnosed as port-site implantation metastasis in clinical practice. a challenge that is universally present in the field of peritoneal metastasis diagnosis and treatment, and is not a rare phenomenon. Based on this, the following clinical implications are proposed:

First, clinicians and radiologists should strengthen information integration, paying particular attention to the dual trauma history of radiotherapy combined with surgery. And they need to focus on the anatomical correlation between the suspected nodule and the laparoscopic surgical operating port. For patients with suspected peritoneal nodules (especially 1–3 cm nodules) of colorectal cancer, PET-CT should be actively recommended as a supplementary examination if conditions permit. Combined with the characteristics of Hif1-α overexpression and active glucose metabolism in colorectal cancer, it can significantly improve the accuracy of benign and malignant differentiation. Comprehensive preliminary screening combining tumor markers, symptoms, and imaging-associated signs can help reduce misdiagnosis rates.

Second, MDT is the core mechanism for decision-making regarding atypical peritoneal lesions in high-risk patients. It can integrate multidisciplinary opinions to seek balance between overtreatment and diagnostic delay.

Third, laparoscopic minimally invasive exploration is a reasonable exclusionary diagnostic approach for high-risk staged patients who refuse follow-up. Its dual characteristics of minimally invasive advantages and pathological gold standard can effectively resolve imaging differentiation dilemmas.

Fourth, for port-site nodular lesions detected after laparoscopic colorectal cancer surgery, clinicians should not simply diagnose them as port-site implantation metastasis, but should make a comprehensive judgment combining the interval between surgery and nodule detection, tumor marker levels, and imaging enhancement characteristics.

The diagnosis of peritoneal lesions in colorectal cancer should follow the “clinical-imaging-pathological” multidimensional verification principle. Only by emphasizing integrated analysis of individual characteristics and medical history details can diagnostic accuracy be improved and patient prognosis optimized.

## Data Availability

The original contributions presented in the study are included in the article/Supplementary Material. Further inquiries can be directed to the corresponding author.
